# *In Situ* FT-IR Spectroelectrochemistry Reveals Mechanistic Insights into Nitric Oxide Release from Ruthenium(II) Nitrosyl Complexes

**DOI:** 10.1021/acs.inorgchem.4c03185

**Published:** 2024-10-30

**Authors:** Felipe de Santis Gonçalves, Lucyano J. A. Macedo, Maykon L. Souza, Nicolai Lehnert, Frank N. Crespilho, Antonio C. Roveda Jr, Daniel R. Cardoso

**Affiliations:** † São Carlos Institute of Chemistry, University of São Paulo, São Carlos 13560-970, SP,Brazil; ‡ Brazilian Synchrotron Light Laboratory, Brazilian Center for Research in Energy and Materials, Campinas 13084-971, SP, Brazil; § Department of Chemistry, University of Michigan, Ann Arbor, Michigan 48109, United States

## Abstract

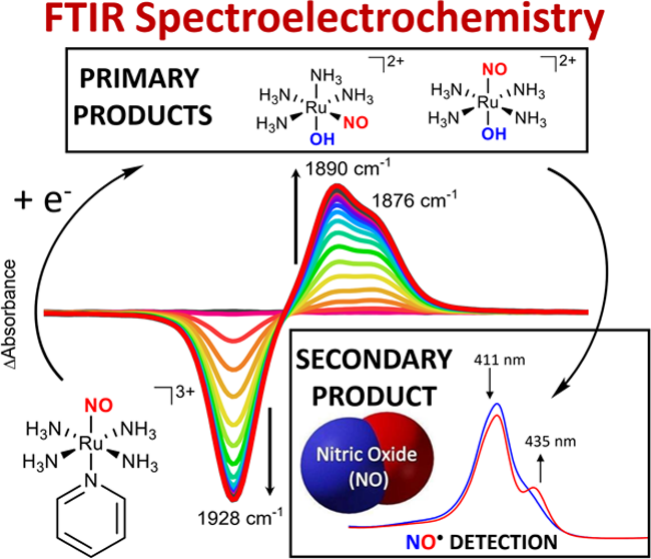

Ruthenium(II) tetraamine nitrosyl complexes with N-heterocyclic ligands are known for their potential as nitric oxide (NO^•^) donors, capable of releasing NO^•^ through either direct photodissociation or one-electron reduction of the Ru(II)NO^+^ center. This study delivers a novel insight into the one-electron reduction mechanism for the model complex *trans*-[Ru^II^(NO)(NH_3_)_4_(py)]^3+^ (RuNOpy, py = pyridine) in phosphate buffer solution (pH 7.4). *In situ* FT-IR spectroelectrochemistry reveals that the pyridine ligand is readily released upon one-electron reduction of the nitrosyl complex, a finding supported by nuclear magnetic resonance spectroscopy (^1^H NMR) and electrochemistry coupled to mass spectrometry (EC-MS), which detect free pyridine in solution. However, direct evidence of NO^•^ release from RuNOpy as the primary step following reduction was not observed. Interestingly, FT-IR results indicate that the isomers of the nitrosyl complex, *cis*-[Ru(NO)(NH_3_)_4_(OH)]^+^ and *trans*-[Ru(NO)(NH_3_)_4_(OH)]^+^, are formed following reduction and pyridine labilization, initiating an outer-sphere electron transfer process that triggers a chain electron transfer reaction. Finally, nitric oxide is liberated as an end product, arising from the reduction of the hydroxyl isomer complexes *cis*-[Ru(NO)(NH_3_)_4_(OH)]^2+^ and *trans*-[Ru(NO)(NH_3_)_4_(OH)]^2+^. This study provides new insights into the reduction mechanism and transformation pathways of ruthenium nitrosyl complexes, contributing to our understanding of their potential as NO^•^ donors.

## Abstract 2

*In situ* FT-IR spectroelectrochemistry reveals insights into the nitric oxide release (NO) mechanism from ruthenium(II) tetraamine nitrosyl complexes. The primary step following the one-electron reduction of the {RuNO}^6^ model complex *trans*-[Ru(NO)(NH_3_)_4_(py)]^3+^ involves the labilization of pyridine, resulting in the formation of two ruthenium nitrosyl isomers, *cis*-[Ru^II^(NO)(NH_3_)_4_(OH)]^+^ and *trans*-[Ru^II^(NO)(NH_3_)_4_(OH)]^+^, followed by the dissociation of NO. Our proposed mechanism offers valuable guidance for designing N-heterocyclic ligands with substituents, potentially broadening the biological applications of these complexes.

## Introduction

Nitric oxide (NO^•^) is an endogenously generated signaling agent that plays critical roles in mammalian metabolism, including vasodilation, neurotransmission, and immune response.^1−3^ These properties have sparked interest in developing compounds capable of controlled NO^•^ release. Examples of NO donors include organic compounds such as nitrates^4^ and diazeniumdiolates,^5,6^ as well as coordination compounds like sodium nitroprusside (SNP).

Sodium nitroprusside ([Fe(CN)_5_NO]^2–^) is a classic example of an NO donor, first reported by Playfair in 1849.^7−9^ This compound delivers NO^•^ through either photochemical or electrochemical activation.^10,11^ However, SNP has some drawbacks as an NO^•^ donor: (i) the Fe^II^NO^•^ complex also labilizes cyanide,^10^ and (ii) modulating the rate of NO^•^ delivery is challenging. The cyanide release issue can be mitigated by the administration of hydroxylamine or sodium thiosulfate.^12,13^ The second issue is particularly significant because the physiological impact of NO^•^ is determined by its local concentration.^14^ Therefore, finding ways to control the NO^•^ delivery without these limitations is a key focus in the development of novel NO donors based on coordination chemistry.

Given the unique properties of NO^•^, researchers were motivated to develop new molecules and materials capable of releasing NO^•^ in a safe and controllable manner.^15−23^ Ruthenium (Ru) complexes have been a subject of extensive study in this pursuit.^24−30^ Ru(II) complexes form stable coordination compounds with NO as an axial ligand and can be formally described as Ru^II^NO^+^ moiety.^31^ Notably, these complexes release NO^•^ upon one-electron reduction, making them promising candidates for controlled NO^•^ delivery.^26,32−35^ Achieving distinct local concentrations of NO^•^ requires the ability to modulate the reactivity of the NO ligand. This modulation can be achieved by modifying the other ligands in the coordination sphere of the Ru complexes. In this regard, a series of promising complexes of the type *trans*-[Ru(NO)(NH_3_)_4_(L)]^*n*+^ has been developed, where L represents N-heterocyclic ligands.^26,31,36^ The established synthetic method for these complexes allows for the preparation of various compounds by simply integrating different N-heterocycles, resulting in a broad spectrum of complexes with differing coordinated NO^+^ ligand reactivity.^36,37^ The electronic properties associated with these N-heterocyclic ligands influence the reactivity of the coordinated NO ligands.^26^ Moreover, due to the high stability and solubility of these nitrosyl complexes, studies have also been conducted incorporating them into materials.^24,25,38^

Structurally, *trans*-[Ru(NO^+^)(NH_3_)_4_(L)]^*n*+^ complexes are octahedral with four equatorial ammines coordinated to the Ru(II) center. These ammines are considered innocent ligands, *i*.*e*., not actively participating in substitution reactions.^26,31^ Along the axial *z*-axis, the NO^+^ is coordinated *trans* to the N-heterocycle. These nitrosyl complexes exhibit remarkable solubility and stability in aqueous solutions,^36,37^ with reduction potentials ranging from *E*_(NO+/NO•)_^0^ = −0.298 V (L = imidazole, coordinated by the C), up to *E*_(NO+/NO•)_^0^ = 0.112 V (*vs* NHE) (L = pyrazine). They are susceptible to reduction by a wide range of biomolecules making them attractive candidates for controlled release of NO^•^ in biological media.^26,31,39−41^ The literature describes the nitrosyl complexes as NO^•^ donors following a one-electron reduction process, which produces the respective aqua-complexes, following eqs 1 and 2:^26,42^
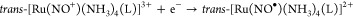
1
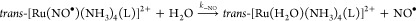
2

The release rate constants (*k*_–NO_) for NO^•^ from these complexes are reported in a range from 0.025 s^–1^ (for L = nicotinamide) to 4.0 s^–1^ (for L = imidazole).^31^ Density Functional Theory (DFT) calculations have indicated that the Lowest Unoccupied Molecular Orbitals (LUMOs) are the antibonding combinations of the Ru (dπ) and NO^+^ (π*) π-backbonding orbitals, with a dominant NO^+^ (π*) contribution.^43^ Consequently, it has been suggested that the initial reduction of these Ru(II)NO^+^ moieties occurs on the NO ligand, leading to the formation of the Ru^II^NO^•^ as an intermediate complex. This proposal has been experimentally supported by Electron Paramagnetic Resonance (EPR) for ruthenium(II) tetraamine nitrosyl complexes.^26,44,45^ Also, FT-IR spectroscopy shows a drastic drop in the N–O stretching frequency upon reduction of the complex.^45^ As a result, the reduced affinity of the NO^•^ ligand for the Ru(II) center facilitates the dissociation of NO^•^.^45^

The generation of the Ru^II^NO^•^ intermediate is a crucial step leading to the labilization of NO^•^. This process can be monitored using various techniques, such as infrared spectroscopy^38^ and electrochemical techniques (*E*^0^ NO^+^/NO^•^ = ∼0.7 V (*vs* SCE)).^37,46^ The resulting aqua complex can be identified using UV–vis spectroscopy, which monitors the formation of *trans*-[Ru(H_2_O)(NH_3_)_4_(L)]^2+^ (eq 2), typically showing absorptions in the 350–450 nm region.^31,36^

In our research, we explored the *in situ* FT-IR spectroelectrochemistry of the complex *trans*-[Ru(NO)(NH_3_)_4_(py)]^3+^ (py = pyridine) and discovered results that contradict the existing literature.^26,31,36,37^ The expected outcome of the electrochemical reduction of *trans*-[Ru(NO)(NH_3_)_4_(py)]^3+^ is described by eqs 1 and 2, where *trans*-[Ru(NO)(NH_3_)_4_(py)]^3+^ would be reduced to generate the respective Ru^II^NO^•^ complex, followed by NO^•^ release. However, by applying a cathodic potential, our results show processes unrelated to the NO^•^ liberation in the initial steps of the reaction. This discrepancy prompted a re-evaluation of the NO^•^ labilization process from the Ru(II) nitrosyl complexes. We studied this reaction using the model complex *trans*-[Ru(NO)(NH_3_)_4_(py)]^3+^ (RuNOpy) in aqueous solution primarily through FT-IR spectroelectrochemistry, along with complementary techniques such as ^1^H NMR spectroscopy and *in situ* electrochemistry coupled to mass spectrometry.

## Experimental Section

### Materials and Methods

#### Reagents and Solutions

All chemicals were purchased from Sigma-Aldrich, Fluka, Honeywell, or Merck and used without further purification. Ruthenium(III) chloride hydrate (Strem Chemicals) served as the synthetic precursor for the investigated ruthenium complexes. High-purity argon (99.998%) from White Martins was used to maintain an anaerobic atmosphere in all experiments. Unless stated otherwise, all experiments were conducted in a phosphate buffer medium (0.1 mol L^–1^, μ = 0.262 mol L^–1^, pH = 7.4 ± 0.1) prepared using high-purity water (18.2 MΩ cm) from a Milli-Q purification system (Millipore, Bedford, MA). For solutions in D_2_O (Sigma-Aldrich 99.9%), the pH was adjusted by adding 0.4 units to the value recorded by the pH meter.^47^

#### Apparatus and Methods

Infrared spectra were acquired using a Shimadzu FTIR spectrophotometer, model IRAffinity-1. Samples were dispersed in KBr pellets and scanned in the range of 4000–400 cm^–1^, with 32 scans and a resolution of 4 cm^–1^. Attenuated total reflectance (ATR) FT-IR spectra were recorded using the corresponding accessory, using a ZnSe crystal as ATR element.

Cyclic voltammetry was conducted using a Princeton Applied Research Polarographic Analyzer (Model 264A). A three-electrode cell was employed, consisting of: (i) a gold disc encapsulated in a Teflon cylinder (working electrode); (ii) a platinum wire (auxiliary electrode); (iii) Ag/AgCl saturated electrode as the reference. The complex concentration was *C*_Ru_ = 5 × 10^–3^ mol L^–1^.

*In situ* infrared spectroelectrochemistry experiments were conducted using a three-electrode cell controlled by a potentiostat Autolab PGSTAT128N connected to an infrared spectrometer Bruker Vertex 70v. A gold disk served as the working electrode, while a platinum wire and Ag/AgCl saturated electrode were used as the auxiliary and reference electrodes, respectively. The experimental procedure closely followed a previously reported method (Figure S1 illustrates our experimental setup).^48^ The gold disk was positioned to face a CaF_2_ window for the incidence of the infrared beam and subsequent reflection, enabling the analysis of chemical changes induced by the application of an electrochemical potential. All spectra presented are the result of subtracting the spectrum obtained at *E* = +0.25 V *vs* Ag/AgCl saturated (applied for 60 s before acquisition). A total of 32 interferograms per spectrum were accumulated using the HgCdTe (MCT) detector, which was cooled with liquid nitrogen (77 K). The interferometer operated at 160 kHz with a spectral resolution of 4 cm^–1^. Each FT-IR spectrum was recorded at 3.5 s time intervals.

UV–vis spectra were recorded on a Shimadzu UV-2600 Spectrophotometer, using a 1 cm path-length quartz cell. Nitric oxide detection experiments were conducted using the Co^II^(TPPS) solution method^49^ in a cuvette and a two-vial system. Vial 1 contained an ascorbic acid solution (n_asc_ = 1.0 × 10^–7^ mol), while vial 2 held the Ru(II) nitrosyl complex solution (n_Ru_ = 2.5 × 10^–6^ mol). The quartz cuvette containing the solution of Co^II^(TPPS) was placed in the sample holder of the UV–vis spectrophotometer. The system was purged with argon (99.998%) for at least 60 min. The ascorbic acid solution was transferred via cannula to the complex solution, reducing the ruthenium complex. Nitric oxide released from the complex was transported via cannula to the Co^II^(TPPS) solution, forming the Co^III^(TTPS)(NO) complex. The NO yield was determined by the ΔAbs of the Co^II^(TPPS) complex (λ_max_ = 411 nm; log ε_411_ = 3.14).^50^ Kinetic experiments were performed in the same manner, monitoring the signal of Co^III^(TTPS)(NO) (λ_max_ = 435 nm) every 1 s.

^1^H and ^15^N Nuclear Magnetic Resonance (NMR) spectra were recorded on an Agilent 500 MHz NMR Spectrometer (Model 500/54 Premium Shield) using an OneNMR probe and the Agilent presat pulse sequence. For internal reference, 3-(Trimethylsilyl)propionic-2,2,3,3-d4 acid sodium salt (TMSP-*d*_4_) was used.

Electron Paramagnetic Resonance (EPR) experiments were performed on a Bruker EMX plus X-band equipment at 77 K (liquid nitrogen) and using a 4104TM/1006 cavity.

The exhaustive electrolysis setup consisted of an electrochemical cell with three electrodes controlled by a potentiostat Autolab PGSTAT204. A gold mesh served as the working electrode, while platinum wire and Ag/AgCl_sat_ were used as the auxiliary and reference electrodes, respectively. Experiments were conducted at *T* = 293 ± 4 K with constant stirring. A potential of *E* = −0.30 V vs Ag/AgCl_sat_ was applied for 1 h.

The online Electrochemistry Coupled to Mass Spectrometry (EC-MS) setup comprised a custom-built electrochemical cell linked to a mass spectrometer, following a methodology akin to prior reports.^51^ A detailed description of the EC-MS setup is provided in Figure S2 (Supporting Information). In a typical experiment, the volatile and gaseous products formed during the electrochemical reactions are detected immediately. The potential is controlled by an Autolab PGSTAT204 potentiostat. The working electrode/interface consists of a gold foil with a series of small holes and a PTFE membrane (Gore-Tex, 0.02 μm pore size and 50 μm thickness) underneath, placed inside a homemade holder (PEEK), which is screwed to the stainless-steel tube that is the probe to the mass spectrometer. Platinum plates and Ag/AgCl_sat_ were used as auxiliary and reference electrodes, respectively. The mass spectrometer OmniStar (Pfeiffer Vacuum) was used to analyze the ionic currents of mass/charge (*m*/*z*) ratios of volatile and gaseous species throughout the electrochemical experiment. The monitored *m*/*z* were 52 and 79, for fragments of pyridine, and *m*/*z* = 30 for nitric oxide.

#### Theoretical Calculations

Density Functional Theory (DFT) calculations were performed using Gaussian 16 software.^52^ The initial structure of the RuNOpy complex was obtained from the crystallographic data (Cambridge Crystallographic Data Centre (CCDC); Deposition Number: 1948978), by removing the water molecule and sulfate and chloride ions from the structure. The initial structures of *cis*-RuNO(OH) and *trans*-RuNO(OH) were obtained by replacing the pyridine ligand from the RuNOpy structure with hydroxide. All calculations were performed without considering any effect from solvents, using the Becke’s 1988 exchange functional^53^ and the gradient corrections of Perdew, along with his 1981 local correlation functional P86 (BP86).^54^ Atoms in the structure for all calculations were treated using the triple-ζ polarized basis set of Alrichs and co-workers (def2-TZVP).^55,56^

#### Synthesis of the Complexes

Complexes [Ru(NH_3_)_5_Cl]Cl_2_, *trans*-[Ru(SO_2_)(NH_3_)_4_(Cl)]Cl, *trans*-[Ru(SO_4_)(NH_3_)_4_(py)]Cl, and *trans*-[Ru(NO^+^)(NH_3_)_4_(isn)](BF_4_)_3_ were synthesized using procedures described in the Literature.^36,57^ Complexes were identified and characterized using UV–vis, FT-IR, ^1^H NMR (for the Ru(II) complexes), and cyclic voltammetry techniques, following procedures reported in the literature.^36,57−59^

##### *trans*-[Ru(NO^+^)(NH_3_)_4_(py)](PF_6_)_3_

(RuNOpy). *trans*-[Ru(SO_4_)(NH_3_)_4_(py)]Cl (90 mg) (2.4 × 10^–4^ mol) is solubilized in 4.0 mL of an argon-saturated HPF_6_ solution at pH = 3.0. The solution is reduced using zinc Amalgam (ZnHg, 5 pieces of 2–14 mesh) for 1 h, resulting in the *trans*-[Ru(H_2_O)(NH_3_)_4_(py)]^2+^ complex. The solution is transferred via cannula to an argon-saturated HPF_6_ 5.0 mol L^–1^ (2.0 mL), followed by the addition of 130 mg of NaNO_2_ (1.9 × 10^–3^ mol) under vigorous stirring. The resulting orange solution is allowed to react for another hour, in argon stream. After this, 140 mg of NH_4_PF_6_ (8.6 × 10^–4^ mol) is added to the solution, followed by the addition of 50 mL of ethanol. A salmon-colored solid is obtained, which is then filtered and washed with cold ethanol, dried and stored in vacuum, in the absence of light. Yield: 56–68%. The ^15^N-labeled *trans*-[Ru(^15^NO^+^)(NH_3_)_4_(py)](PF_6_)_3_ was synthesized using Na^15^NO_2_ (98 atom % ^15^N, Sigma-Aldrich).

##### *trans*-[Ru(H_2_O)(NH_3_)_4_(py)]^2+^

*trans*-[Ru(SO_4_)(NH_3_)_4_(py)]Cl (2.0 mg) (5.3 × 10^–6^ mol) was solubilized in 1.0 mL of phosphate buffer solution (0.1 mol L^–1^) prepared in D_2_O (Sigma-Aldrich 99.9%), degassed using Argon (99.998%). This solution was transferred via cannula to a vial containing a single Zinc Amalgam (ZnHg). The solution was allowed to react for 35 min, under Argon stream, and was subsequently transferred to an NMR tube with cap and septa, using the Argon flux, and was directly used for ^1^H NMR analysis (Figure S14).

##### *trans*-[Ru(NO^+^)(NH_3_)_4_(H_2_O)](TFMS)_3_

*trans*-[Ru(NO^+^)(NH_3_)_4_(isn)](BF_4_)_3_ (70 mg) (1.2 × 10^–4^ mol) is solubilized in 4.0 mL of phosphate buffer (0.1 mol L^–1^) μ = 0.262 mol L^–1^. The solution is heated to 50 °C for 24 h. This procedure induces the dissociation of the isonicotinamide, which is substituted by a water molecule.^58^ To this solution, 1.0 mL of trifluoromethanesulfonic acid (5.0 mol L^–1^) is added. The final solution is refrigerated at 5 °C. After 12 h, the solution is brought to a Schlenk line, and the volume is reduced under vacuum until yellow crystals are observed. This complex was collected by filtration, and the solid was dried and stored under vacuum and protected from light. Yield: 31%.

##### Co^II^(TPPS)

Cobalt(II) chloride hexahydrate (10 mg) (4.2 × 10^–5^ mol) was solubilized in 2.0 mL of methanol. To this solution, TPPS (4,4′,4″,4‴-(Porphine-5,10,15,20-tetrayl)tetrakis(benzenesulfonic acid) tetrasodium salt hydrate) (10 mg) (9.6 × 10^–6^ mol) was added. The reaction was allowed to react for 1 h. The Co^II^(TPPS) solid is collected by filtration, washed with cold methanol (15 mL), dried and stored under vacuum in the absence of light. Experimental details about NO detection using Co^II^(TPPS) are given in the Supporting Information (Figure S3).

## Results and Discussion

**Figure 1:**
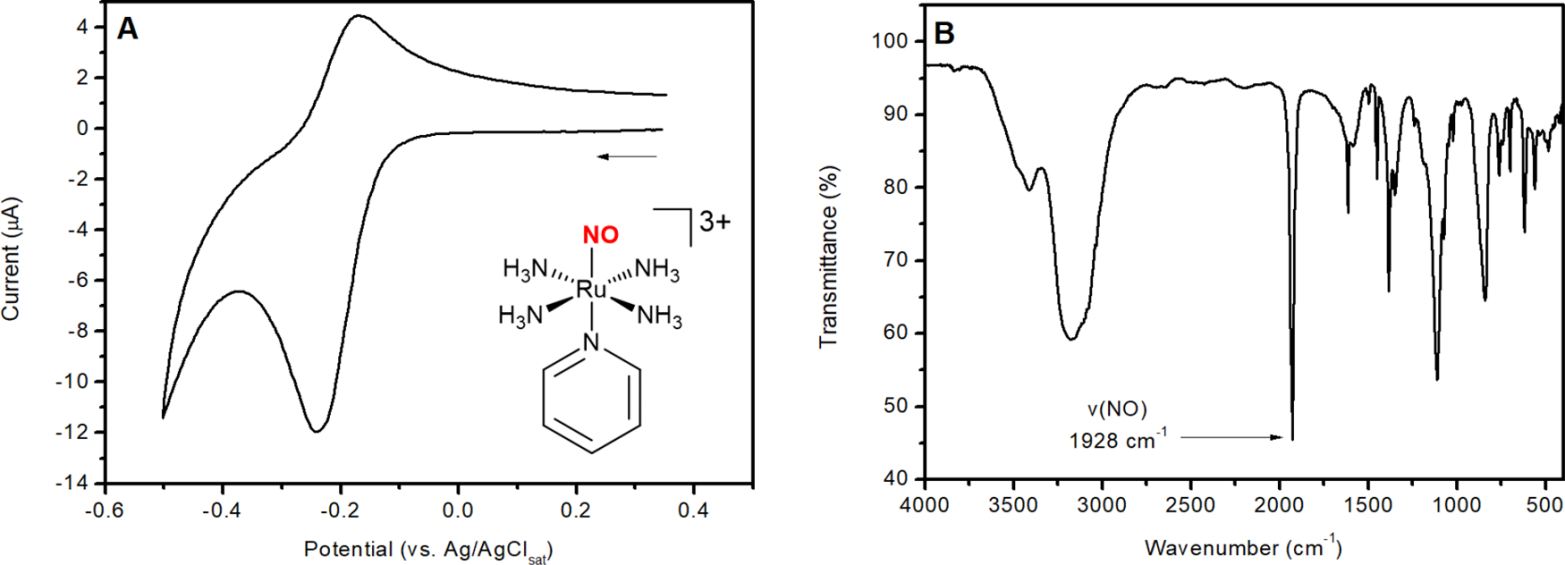
(A) Cyclic voltammogram of RuNOpy (5 × 10^–3^ mol L^–1^) in phosphate buffer solution (pH = 7.4 ± 0.1; μ = 0.262 M). Scan rate: 100 mV s^–1^. (B) Infrared spectrum of RuNOpy, in KBr pellet.

The complex under investigation, RuNOpy, is a low-spin Ru(II) complex with a coordinated NO^+^ ligand, making it EPR silent. Cyclic voltammetry experiments of RuNOpy (Figure 1A) demonstrated a reductive process between +0.4 and −0.4 V (vs Ag/AgCl_sat_), owing to the Ru^II^NO^+^/Ru^II^NO^•^ pair,^31^ as shown in eq 1. Of note, all the redox potentials reported in the “Results and Discussion” section are referenced vs. Ag/AgCl_sat_. The infrared spectrum of RuNOpy shows a clear signal at 1928 cm^–1^ (Figure 1B), which corresponds to the ν(NO) vibration. Other RuNO complexes with N-heterocyclic ligands *trans-*positioned to NO^+^ typically exhibit bands in the 1940–1920 cm^–1^ range,^31,36,37,60^ which is characteristic of this class of complexes and supports the assigning as a Ru^II^NO^+^ moiety, with a linear Ru–N–O bond angle.^26,31,36,42^

Spectroelectrochemistry offers real-time monitoring of changes in nitrosyl complexes when a specific redox potential is applied to the system. This is accomplished by tracking negative changes in the difference of absorbance, which indicate the consumption of reactants, and positive changes in the difference of absorbance, which correspond to product formation. The experiments were carried out in D_2_O solutions (phosphate buffer at pH 7.4) with a complex concentration of C_Ru_ = 5 × 10^–3^ M. All potentials are referenced to Ag/AgCl_sat_. The spectroelectrochemical results were compared with the spectrum obtained by applying a potential of E = +0.25 V, at which no electrochemical process occurs (Figure 1A). The formation of the nitro complex *trans*-[Ru(NH_3_)_4_(NO_2_)(py)]^+^ under the experimental conditions (phosphate buffer, pH 7.4) was ruled out since the nucleophilic attack of the hydroxyl ions on the nitrosyl ligand only occurs at pH above 10 (Figures S4 and S5, Supporting Information).

**Figure 2:**
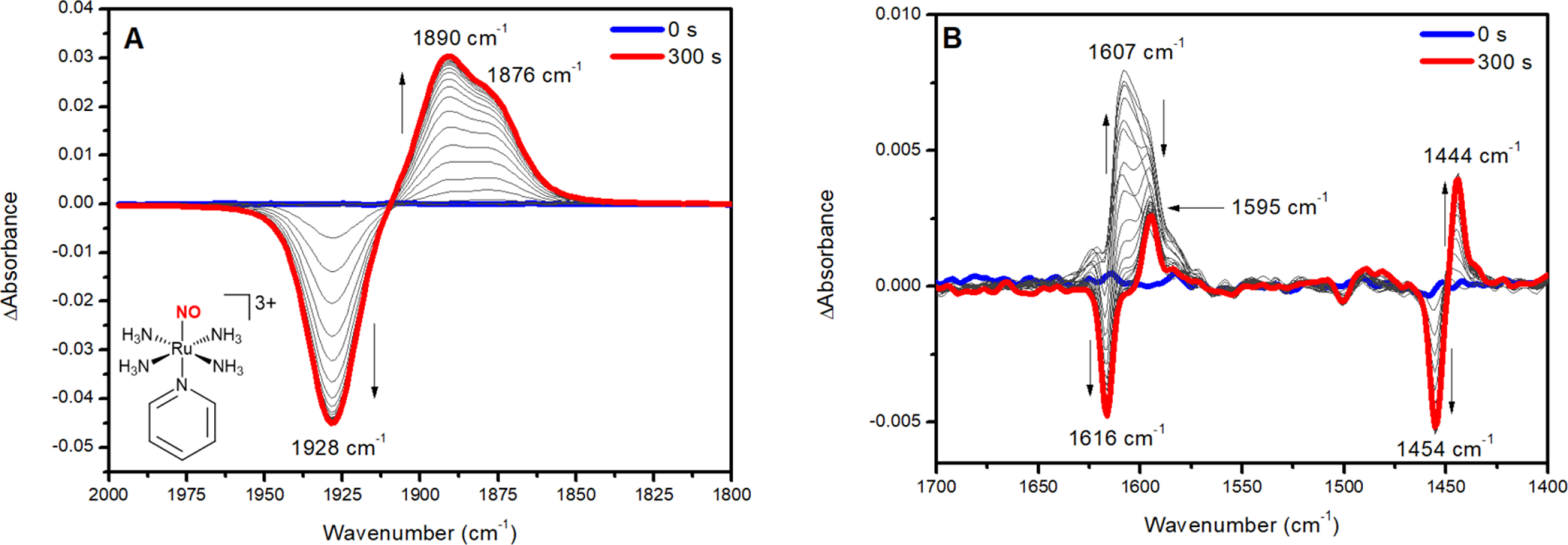
FT-IR spectroelectrochemistry of RuNOpy (5 × 10^–3^ mol L^–1^) upon applying *E* = −0.20 V (vs Ag/AgCl_sat_), in phosphate buffer solution prepared using D_2_O (pH = 7.4 ± 0.1; μ = 0.262 M) (A) Spectral region of 2000–1800 cm^–1^. (B) Spectral region of 1700–1400 cm^–1^. Each FT-IR spectrum was recorded at 3.5 s time intervals.

Figure 2 shows the FT-IR spectral changes of RuNOpy after applying a cathodic potential of *E* = −0.20 V. Figure 2A shows a decrease in intensity of the band at 1928 cm^–1^, which corresponds to the ν(NO) for RuNOpy (Figure 1B), followed by the growth of bands at 1890 and 1876 cm^–1^. Figure 2B shows the spectral region of 1700–1400 cm^–1^ to provide further information. The reduction process involves a decrease in the intensity of signals at 1616 and 1454 cm^–1^, followed by an increase in the intensity of signals at 1595 and 1444 cm^–1^. These signals are attributed to coordinated and labilized pyridine in solution, respectively. This was supported by the FT-IR spectrum of free pyridine under the identical experimental conditions, which shows the same peaks at 1595 and 1444 cm^–1^ (Figure S7). Furthermore, ^1^H NMR and EC-MS studies were carried out, both of which revealed signals attributed to free pyridine after electrolysis of the solution containing RuNOpy (Figures S8 and S9), demonstrating the labilization of pyridine from RuNOpy during electrolysis.

Figure 2B shows an extra absorption band at 1607 cm^–1^, which represents an intermediate species that increases over time, then drops and is not identified in the final spectrum. Time-trace profile data demonstrated a direct correlation between this signal and a decrease in the 1928 cm^–1^ band (Figure S10). Interestingly, the band at 1607 cm^–1^ appears before the bands centered at 1890 and 1876 cm^–1^ rise. The 1607 cm^–1^ band has a 321 cm^–1^ red shift regarding RuNOpy’s ν(NO). In other ruthenium nitrosyl complexes, one-electron reduction of the Ru^II^NO^+^ moiety to Ru^II^NO^•^ species results in similar red-shifts of approximately 300 cm^–1^.^61−66^ We assign the 1607 cm^–1^ band to the complex RuNO^•^py, suggesting a species with NO^•^ still coordinated to the metal center.

To investigate this hypothesis, we synthesized the isotopically substituted compound Ru^15^NOpy. The ν(NO) of Ru^15^NOpy was determined at 1890 cm^–1^ (Figure S11). The reduction process revealed a novel band at 1576 cm^–1^, showing that the 1607 cm^–1^ band is associated with the N–O stretch of the reduced Ru^II^NO^•^ species (Figure S12). Further examination of the 2000–1800 cm^–1^ region with Ru^15^NOpy revealed that all absorption bands in Figure 2A relate to NO species associated with the RuNOpy complex. This is evident from the red shift observed due to ^15^NO isotopic substitution (Figure S11). In addition, the 77K X-band EPR spectrum of the electrolyzed solution was found to be EPR silent, indicating that tetraamine Ru(III) complexes were not formed.^67,68^ This suggests that another reaction mechanism is in operation and that an intermediate Ru^II^NO^+^ aqua complex may be formed.

**Figure 3:**
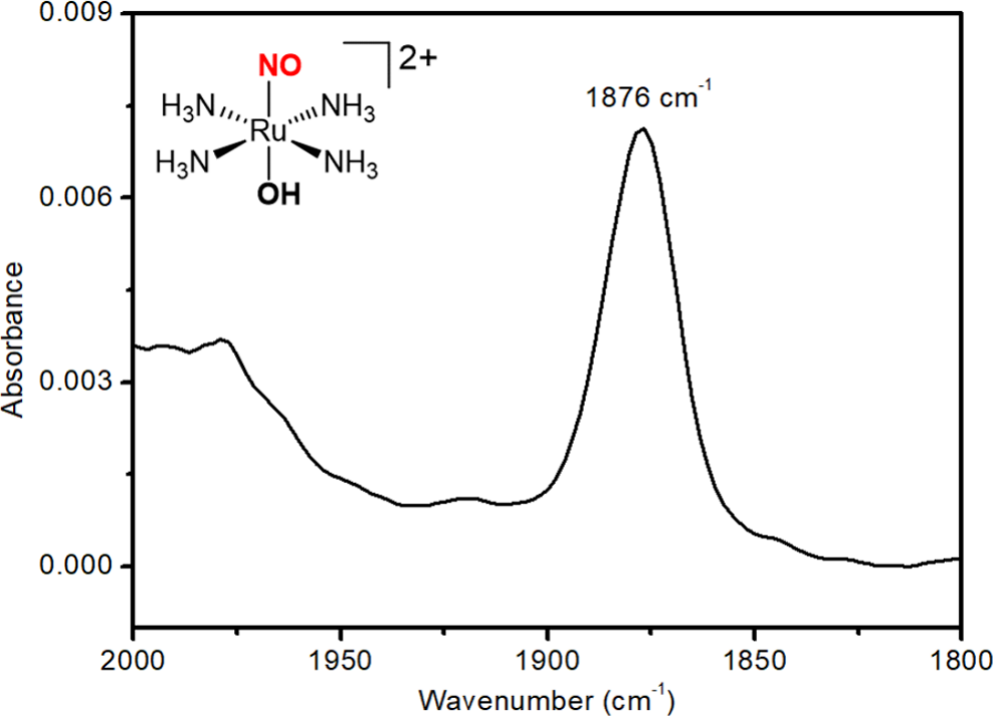
ATR-FT-IR spectrum of *trans*-RuNO(OH) in phosphate buffer solution (pH = 7.4 ± 0.1; μ = 0.262 M).

Although the ν(NO) of the free NO^•^ is reported by FT-IR at 1876 cm^–1^,^69−71^ we verified that after exhaustive purging the product solution with argon, the bands centered at 1876 and 1890 cm^–1^ persisted (Figure S13), suggesting that the band at 1876 cm^–1^ could be assigned to the ν(NO) of the *trans-*RuNO(OH) (Figure 3) (see Figures S8 and S9). Further analysis was carried out using the synthesized complex *trans*-RuNO(OH), which revealed the ν(NO) band at 1876 cm^–1^ (Figure 3), confirming that after one-electron reduction of RuNOpy, one of the intermediate complexes formed is the *trans*-RuNO(OH). The other band centered at 1890 cm^–1^ could be *cis*-RuNO(OH), which results from a *cis–trans* geometrical change that occurs during the electrochemical one electron reduction of the nitrosyl complex.^72^ We attempted to synthesize^73,74^ this *cis-* complex but were unsuccessful in obtaining an isolated pure complex.

**Figure 4:**
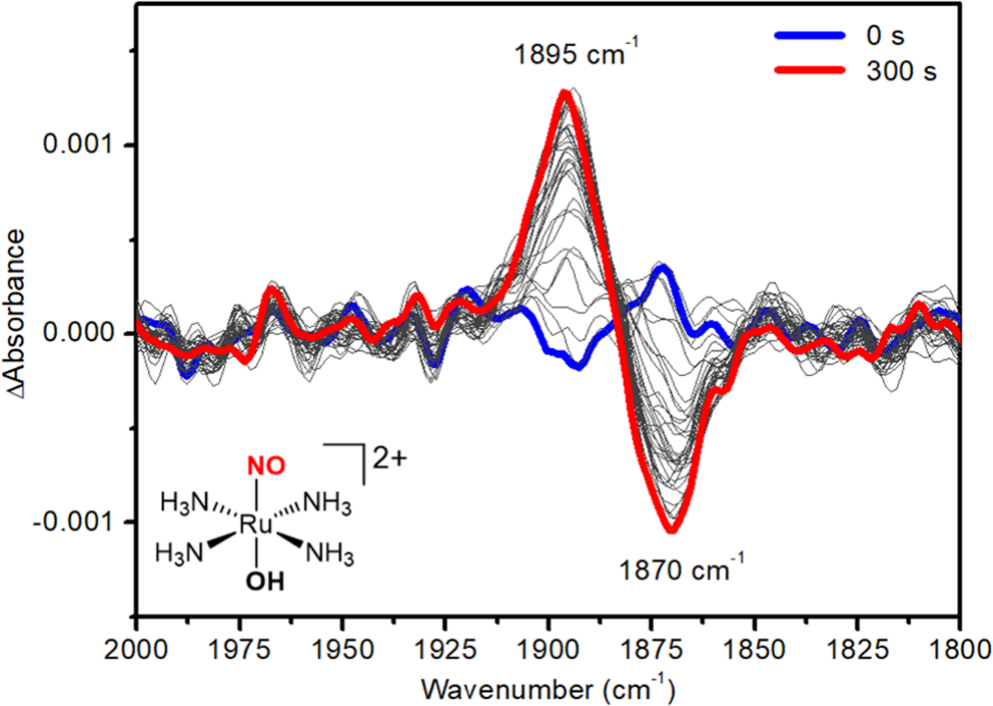
FT-IR spectroelectrochemistry of *trans*-RuNO(OH) (5 × 10^–3^ mol L^–1^) upon applying −0.45 V (vs Ag/AgCl_sat_), in phosphate buffer solution (pH = 7.4 ± 0.1; μ = 0.262 M). Each FT-IR spectrum was recorded at 3.5 s time intervals.

Further exhaustive electrolysis of the *trans-*RuNO(OH) complex at *E* = −0.45 V is shown to decrease the intensity of the band centered around 1870 cm^–1^ (ν(NO)) resulting in the development of the band centered at 1895 cm^–1^. This suggests that after one-electron reduction of *trans*-RuNO(OH), the coordinated NO^•^ ligand induces changes in the coordination sphere, resulting in isomerization to the *cis* complex, *cis-*RuNO(OH), which exhibits a band at 1895 cm^–1^ (Figure 4).^72^ In our system, the ν(NO) at 1890 cm^–1^ is higher than that of *trans*-RuNO(OH) (1876 cm^–1^), and the 14 cm^–1^ difference in ν(NO) is within the published range^73^ and in agreement with DFT calculations (Table S1).

A previous spectroelectrochemistry investigation with an analogous Ru(II) nitrosyl complex, *trans*-[Ru(NO^+^)(NH_3_)_4_(isn)]^3+^ (isn = isonicotinamide),^75^ observed the formation of the same bands at ∼1900 and 1876 cm^–1^ after the electrochemical reduction of this complex, similar to our observations reported here for *trans*-[Ru(NO^+^)(NH_3_)_4_(py)]^3+^ (Figure 2A). The authors did not fully investigate the attribution of these two bands and only assigned the band at 1876 cm^–1^ to a RuNO^•^ species. Here, we show that the band at 1876 cm^–1^ is the ν(NO) of the complex *trans*-[Ru(NO^+^)(NH_3_)_4_(OH)]^2+^ and the band centered at 1890 cm^–1^ is the ν(NO) of the complex *cis-*RuNO(OH) (Figure S15). These results show that both complexes *trans*-[Ru(NO^+^)(NH_3_)_4_(L)]^3+^, in which L = isn or py, produce *trans*-[Ru(NO^+^)(NH_3_)_4_(OH)]^2+^ and *cis*-[Ru(NO^+^)(NH_3_)_4_(OH)]^2+^ after the electroreduction.

**Figure 5:**
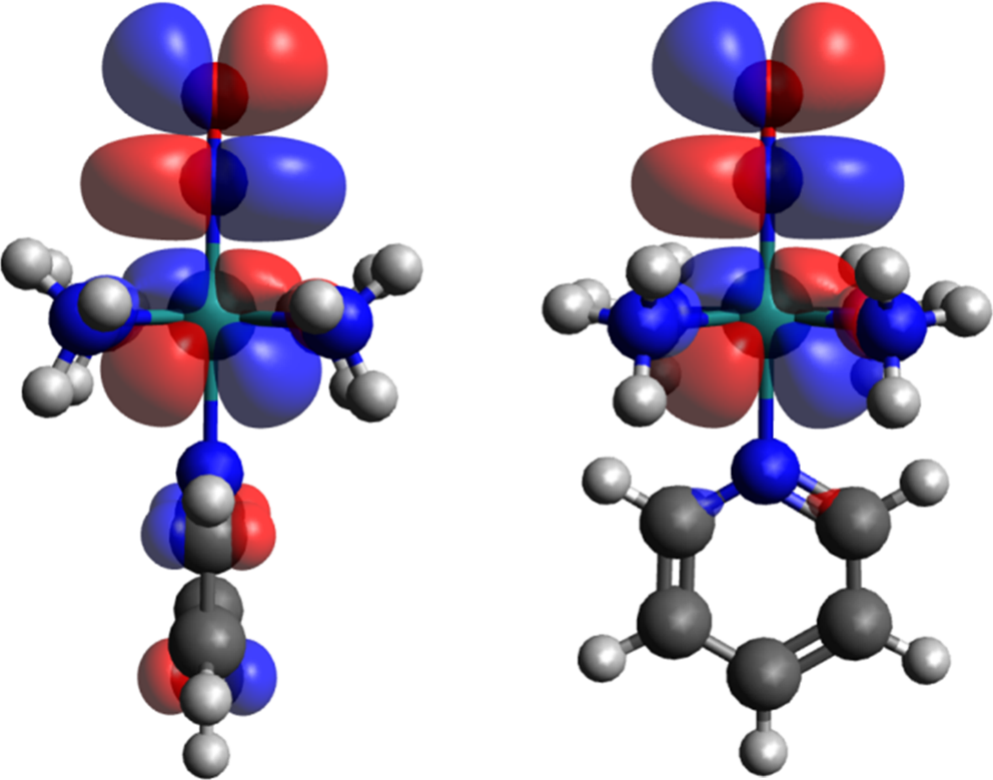
Molecular orbitals LUMO and LUMO+1 for *trans*-[Ru(NO^+^)(NH_3_)_4_(py)]^3+^. Note that in the MO contour plot on the right, the complex is rotated by 90°.

In RuNOpy, the Ru-NO bond is composed of a σ bond Ru ← NO and a backbonding π interaction between d_*xz*_Ru → π_(x)_*NO and d_*yz*_Ru → π_(y)_*NO.^43^ The latter two interactions create the LUMO and LUMO+1 of RuNOpy (Figure 5). Notably, these orbitals have a substantial π*(NO) contribution, as confirmed by DFT and consistent with previously published data.^31^

Reducing RuNOpy causes an electron to occupy the LUMO orbital, resulting in a coordinated NO^•^ signal at 1607 cm^–1^ in the FT-IR spectrum (Figure 2B). The electron in the π* orbital of the NO^•^ ligand alters its acidity. NO^•^ exerts a *trans* influence and effect on the pyridine ligand, causing the pyridine to dissociate.^76,77^ DFT simulations showed an elongation of the Ru–N(pyridine) bond from 2.13 Å for RuNOpy to 2.21 Å for RuNO^•^py.

NO^•^ exerts a *trans* influence and effect on pyridine, causing a substitution reaction with water. Ru(II) ammine substitution reactions are likely to exhibit dissociative characteristics.^78^ Because of ligand labilization, the intermediate in a dissociative substitution process involving a hexa-coordinated complex such as RuNOpy should lead to a penta-coordinated complex. RuNOpy is expected to undergo a dissociative process, resulting in an intermediate similar to the penta-coordinated complex Ru^II^NO^•^.^79−83^ The penta-coordinated transient complex allows water molecules to occupy either the *cis* or *trans* position, forming *cis-*RuNO^•^(OH) and *trans-*RuNO^•^(OH) intermediates which are in a chain reaction converted to *cis-*RuNO(OH) and *trans*-RuNO(OH) in a similar way as previously reported for the complex [Ru(NH_3_)_5_NO]^3+^.^84^ To investigate the hypothesis of a chain reaction in our system, we performed an experiment using Zn(Hg) amalgam as a reductant. This experiment involved transferring a solution of the reduced molecule *cis*/*trans*-RuNO^•^(OH) to another reaction vessel that only contained RuNOpy. The ^1^H NMR spectrum of the final solution (Figure S16) shows that pyridine was quantitatively labilized. Thus, *cis/trans*-RuNO^•^(OH) is shown to be capable to transfer one electron to the RuNOpy species, resulting in pyridine dissociation.

The reduction of the RuNOpy complex promotes competition along the NO^•^-Ru-py axis, with NO^•^ having a significant *trans* effect on the pyridine ligand. To operate as an NO donor, pyridine must stay in the coordination sphere of RuNO^•^py, and a water molecule should then substitute NO^•^, according to eqs 1 and 2. Our results shows that pyridine, instead of the NO^•^ ligand, performs a substitution process with a water molecule generating *cis-*RuNO^•^(OH) and *trans-*RuNO^•^(OH) complexes. RuNOpy complexes can accept an electron, starting the chain reaction, as previously described. This chain reaction continues until the entire RuNOpy complex is consumed. As the reaction develops, the solution eventually contains mostly *cis-* and *trans-*RuNO(OH) complexes, as well as free pyridine. Any residual *cis-*RuNO^•^(OH) or *trans-*RuNO^•^(OH) complexes will enter a critical phase. As the rate of isomerization slows, NO^•^ is labilized from the coordination sphere and then the hydroxo RuNO^•^(OH) complexes function as NO^•^ donors (*k* = 0.04 s^–1^).^23^

**Figure 6:**
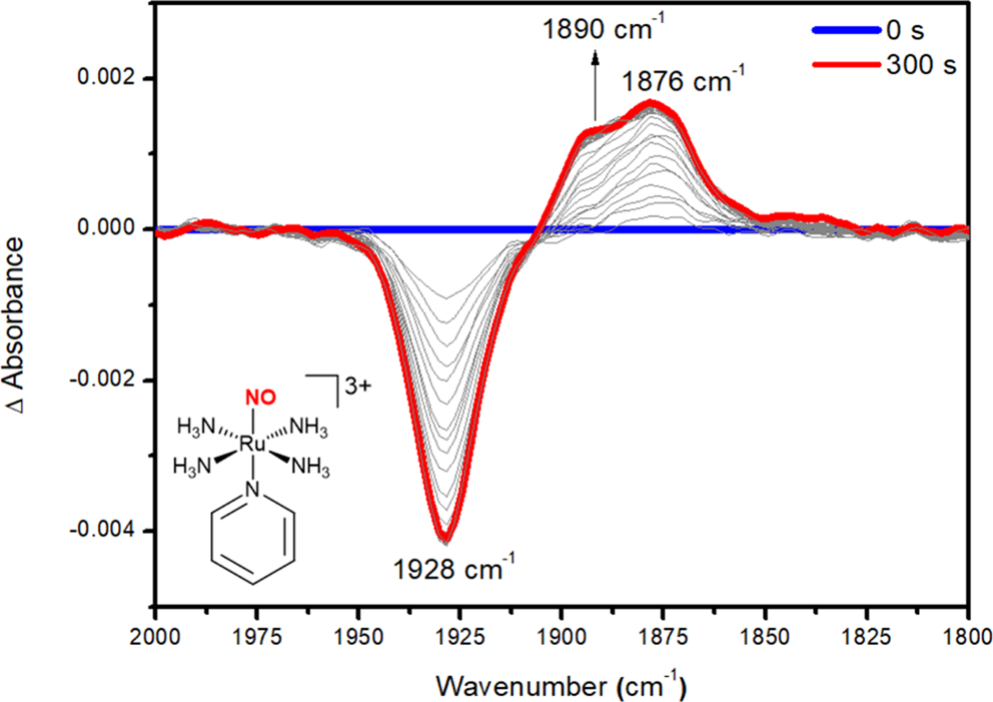
Spectral variations for the reaction between RuNOpy (5 × 10^–3^ mol L^–1^) and ascorbic acid (0.20 mol L^–1^), in phosphate buffer solution (pH = 7.4 ± 0.1; μ = 0.262 M). Each FT-IR spectrum was recorded at 3.5 s time intervals.

To evaluate the chemical reduction of RuNOpy, we reacted the nitrosyl complex with ascorbic acid as a biological reductant. The FT-IR spectroscopic analysis in Figure 6 corroborates that the chemical reduction provides the same products as seen in the electrochemical experiments, namely *cis-*RuNO(OH) and *trans*-RuNO(OH), which suggests a similar reaction mechanism. This finding has significant implications for the biological applications of these nitrosyl complexes, as their capacity to release nitric oxide when reduced is critical. Further research is needed to better understand the aspects that impact their biological function, including NO^•^ release, *trans* ligand behavior, and product reactivity. Furthermore, the observed *trans* ligand labilization opens new possibilities for drug delivery systems. By coordinating specific molecules to the nitrosyl complex, they can be released via electrochemical or chemical reduction, potentially making them accessible to biological reductants and providing a fresh strategy for designing drug-delivery systems.

**Figure 7:**
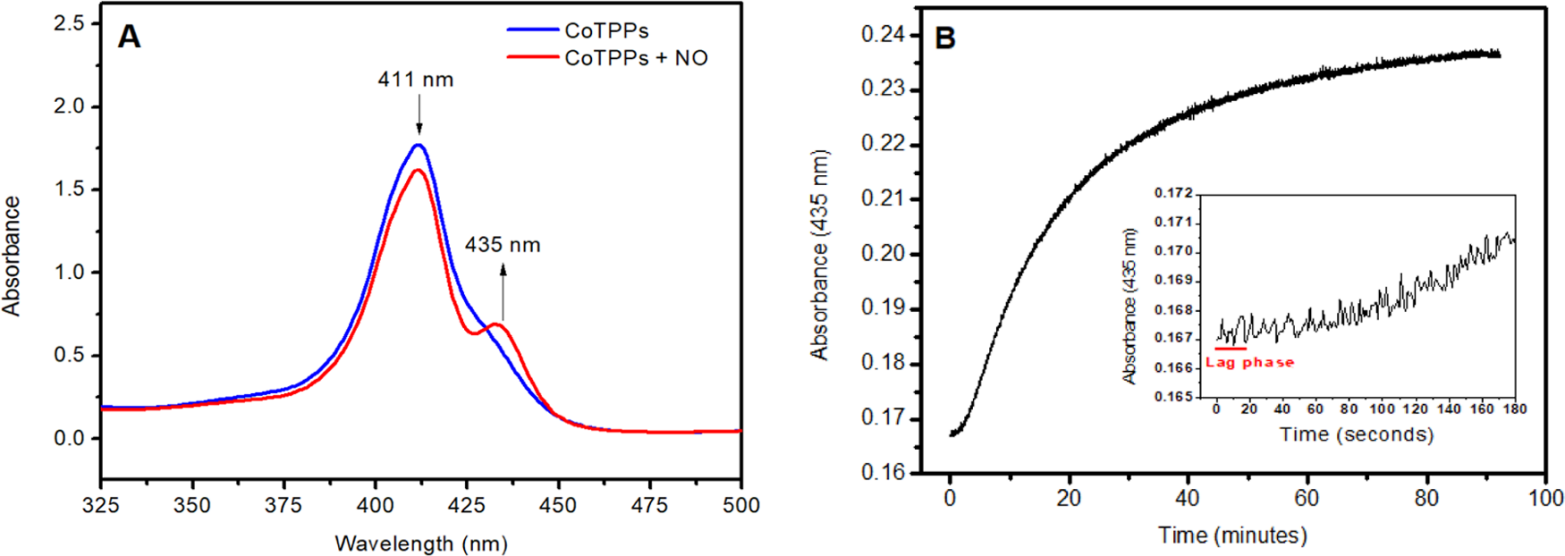
(A) UV–vis spectral variation for Co^II^(TPPS) and Co^III^(TPPS)(NO). NO^•^ is labilized from the reaction between ascorbic acid and RuNOpy. (B) Time-resolved experiment following the band at 435 nm, corresponding to the adduct Co^III^(TPPS)(NO). Inset: Time-resolved result, in the 0–180 s’ range.

Nitric oxide dissociation was further demonstrated by reacting an argon-saturated solution of ascorbic acid with an excess of argon-saturated solution of RuNOpy. This reaction yields quantitatively both *cis*-RuNO^•^(OH) and *trans*-RuNO^•^(OH) as intermediates. In the absence of alternative electron acceptors, these intermediates begin to labilize NO^•^. The NO^•^ labilized by this reaction was transferred–using a positive Argon flux–to a cuvette containing the nitric oxide chemical trap Co^II^(TPPS) (*k*_on_ = 1.9 × 10^9^ M^–1^ s^–1^) dissolved in phosphate buffer pH 7.4 (Figure S3, Supporting Information).^49^Figure 7A depicts the spectral changes in the reaction cuvette containing the complex Co^II^(TPPS), highlighting the emergent band at 435 nm caused by the formation of the complex Co^III(^TPPS)(NO).^85^Figure 7B shows a time-trace profile at 435 nm, indicating a lag phase of tens of seconds. This suggests that all RuNOpy is consumed before NO^•^ is released from *cis*-RuNO^•^(OH) and *trans*-RuNO^•^(OH) products. From data in Figure 7A, the amount of NO^•^ labilized was determined to be 8.6 × 10^–8^ mol (reaction yield of 86%).

**Figure 8:**
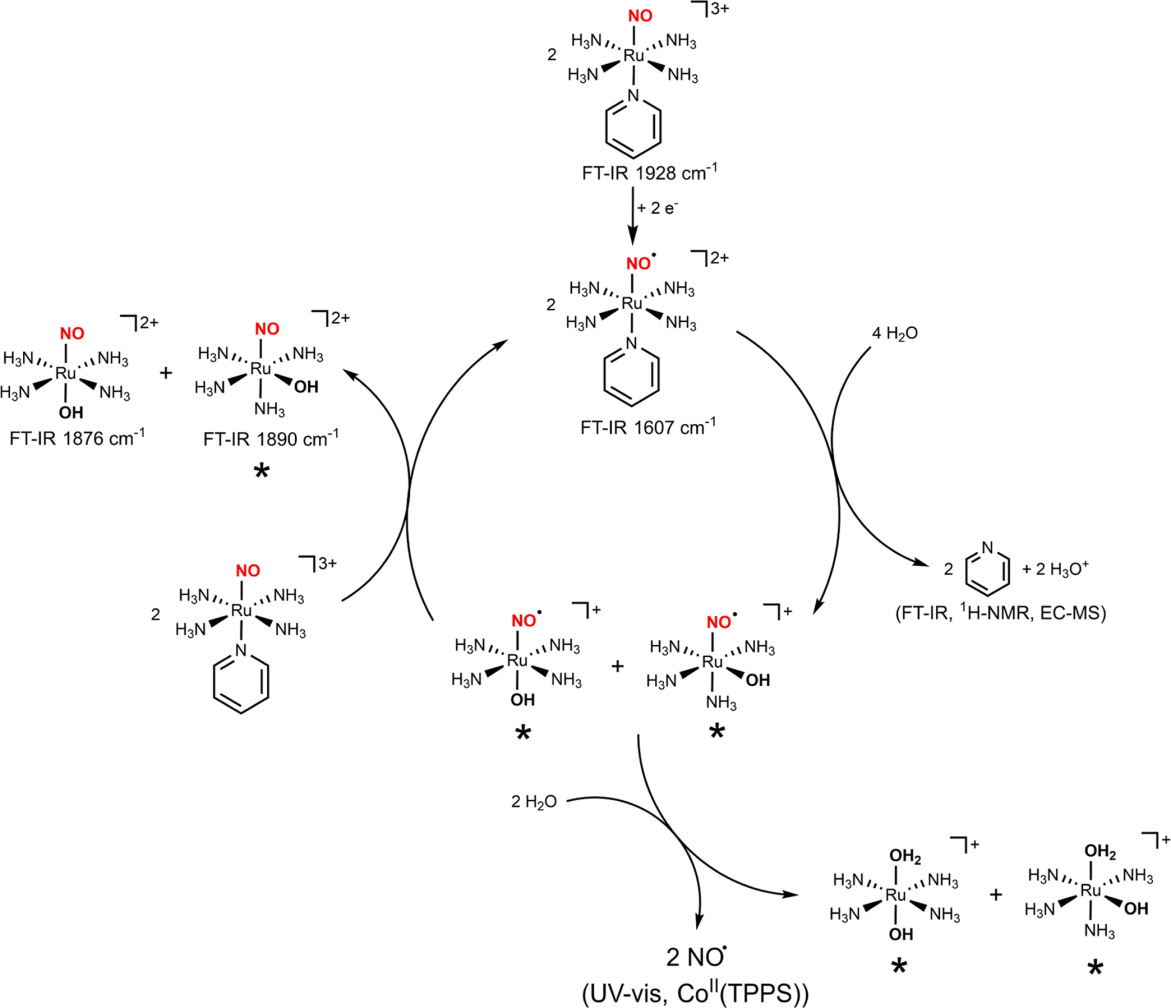
Proposed route for the generation of the nitrosyl hydroxo isomer complexes, and NO release (the complexes marked with an asterisk were not isolated and were annotated based on spectroscopic data and DFT analysis).

Figure 8 summarizes the proposed pathway for the reduction of RuNOpy. Both chemical and electrochemical reductions of RuNOpy primarily result in the labilization of the pyridine ligand, leading to the formation of *cis*-RuNO(OH) and *trans-*RuNO(OH) complexes. The precursor complex, RuNOpy, is subsequently consumed in a chain reaction involving *cis*-RuNO^•^(OH) and *trans*-RuNO^•^(OH) complexes. As most of the RuNOpy is consumed in this chain reaction, the intermediate complexes *cis-*RuNO^•^(OH) and *trans*-RuNO^•^(OH) ultimately release NO^•^. The proposed complexes formed after NO^•^ release are the Ru(II) aqua/hydroxo *cis-* and *trans*-[Ru(H_2_O)(NH_3_)_4_(OH)]^+^. It is important to reinforce that no evidence of Ru(III) was observed by EPR spectroscopy.^67,68^ While some Ru(III) centers may be EPR silent, even at liquid nitrogen temperatures (77 K),^86,87^ analogous tetraamine Ru(III) centers are typically not EPR silent, supporting our mechanism.^67,68^

## Conclusions

In conclusion, our findings reveal that after the chemical and electrochemical reaction of RuNOpy an outer-sphere electron transfer pathway occurs that initiates a chain reaction, resulting in the formation of the hydroxyl isomer products *cis*-[Ru(NO^+^)(NH_3_)_4_(OH)]^2+^ (*cis*-RuNO(OH)) and *trans*-[Ru(NO^+^)(NH_3_)_4_(OH)]^2+^ (*trans*-RuNO(OH)). Notably, NO^•^ is produced because of electron transfer during this chain reaction. This unexpected series of events challenges long-held assumptions about the mechanism of NO^•^ release from ruthenium nitrosyl complexes, opening new avenues for understanding their reactivity. Furthermore, our results provide insights for designing new N-heterocyclic ligands with substituents that can broaden the biological applications of these complexes.

## Supporting Information Available

Schematics diagram of the spectroelectrochemical and EC-MS cell, ATR-IR spectra, ^1^H NMR spectra, EC-MS data, schematic of the experiment of NO detection, discussion of the nucleophilic attack of hydroxyl ions, UV–vis spectra, FT-IR spectroelectrochemical data, and experimental and calculated FT-IR frequency for (NO) for precursors complex and products, and optimized coordinates for structure of *trans*-[Ru(NO^+^)(NH_3_)_4_(py)]^3+^, *trans*-[Ru(NO^•^)(NH_3_)_4_(py)]^2+^, *cis*-[Ru(NO^+^)(NH_3_)_4_(OH)]^2+^ and *trans*-[Ru(NO^+^)(NH_3_)_4_(OH)]^2+^ (PDF)
