# Supplementary material for: In Situ FT-IR Spectroelectrochemistry Reveals Mechanistic Insights into Nitric Oxide Release from Ruthenium(II) Nitrosyl Complexes
Source: Inorg Chem. 2024 Oct 30;63(45):21387–96. doi: 10.1021/acs.inorgchem.4c03185 (PMC11558665; doi:10.1021/acs.inorgchem.4c03185)
Supplement: Supplementary file 1 — ic4c03185_si_001.pdf [file ic4c03185_si_001.pdf]

SUPPORTING INFORMATION (SI)

In Situ FT-IR Spectroelectrochemistry Reveals Mechanistic Insights Into Nitric Oxide Release From Ruthenium(II) Nitrosyl Complexes

Felipede Santis Gonçalves[a], Lucyano J. A. Macedo[a,b], Maykon L. Souza[a], Nicolai Lehnert[c], Frank N. Crespilho[a], Antonio C. Roveda Jr.[a*], Daniel R. Cardoso[a*].

[a]São Carlos Institute of Chemistry, University of São Paulo, São Carlos, SP, Brazil
[b]Brazilian Synchrotron Light Laboratory, Brazilian Center for Research in Energy and Materials, Campinas, SP, Brazil
[c]Department of Chemistry, University of Michigan, United States

*Corresponding Authors:

Antonio C. Roveda Jr. (acroveda@usp.br)
Daniel R. Cardoso (drcardoso@usp.br)

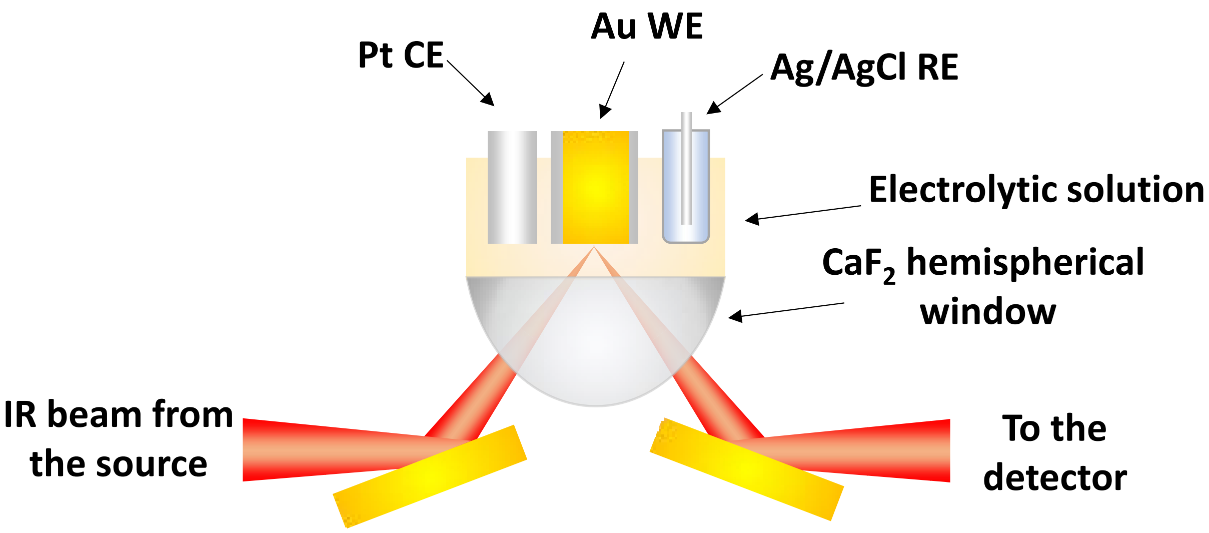

Figure S1. Schematic representation of the spectroelectrochemistry experiment.

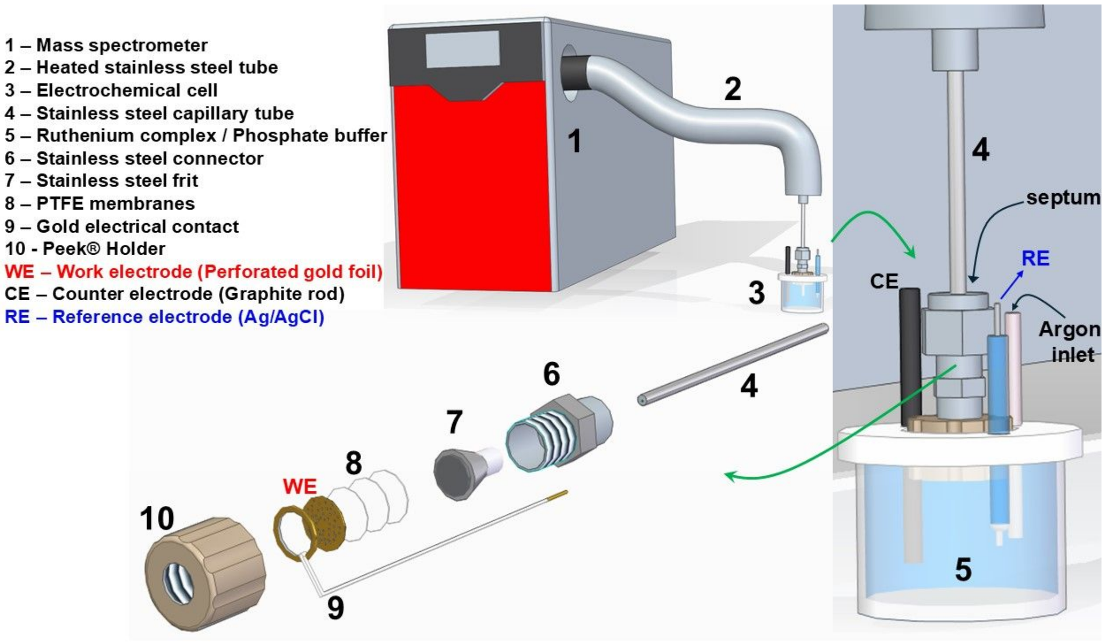

Figure S2. Schematic representation of the electrochemistry coupled to mass spectrometry (EC-MS) setup.

Detection of NO using CO^{II}TPPS

Nitric oxide detection experiments were carried out using the Co^{II}(TPPS) solution method.[1] The system comprises two-vials and a quartz cuvette, as shown in Figure S3. Vial 1 contained an ascorbic acid solution (C = 0.02 mol L^{-1}), while vial 2 held the RuNOpy solution (C_{Ru} = 0.5 x 10^{-3} mol L^{-1}). The quartz cuvette is placed in the sample holder of the UV-Vis spectrophotometer. The system is constantly purged with argon (99.998%). The ascorbic acid solution is then transferred via cannula to the complex solution, reducing the Ru(II) nitrosyl complex. Nitric oxide gas released from the the Ru(II) nitrosyl complex is then transported via cannula to a Co^{II}(TPPS) solution, yielding the Co^{III}(TTPS)(NO) complex. The Co^{III}(TTPS)(NO) complex has an absorption band centered at 435 nm. Kinetic experiments were carried out in the same manner, following the signal at 435 nm every 1 second.

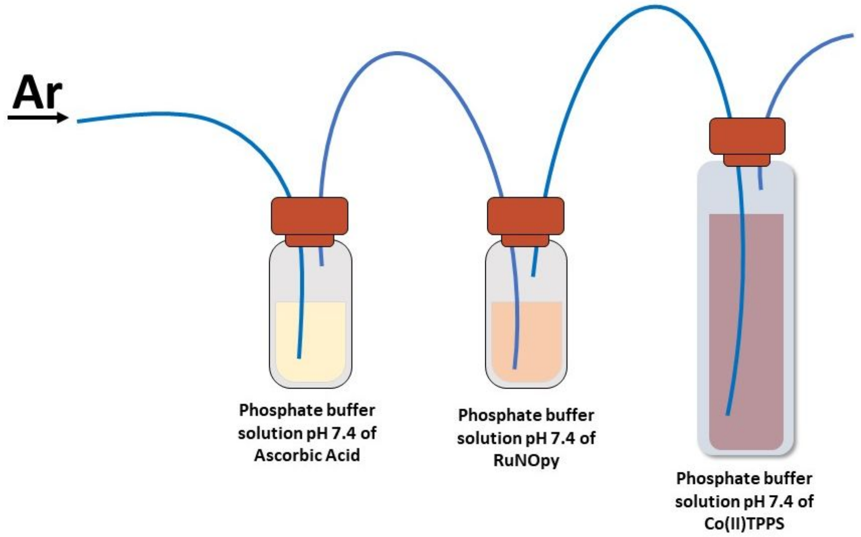

Figure S3. Schematic representation of the detection of NO using Co^{II}(TPPS).

Nucleophilic attack of hydroxyl ions (OH^-) on nitrosyl complexes

Literature reports the nucleophilic attack of hydroxyl ions (OH^-) on nitrosyl complexes, yielding to the corresponding nitro complexes (Ru-NO_2), according to the following general equation:

$$\textit{trans}\text{-}[Ru(NH_3)_4(NO)(L)]^{3+} + 2OH^- \rightleftharpoons \textit{trans}\text{-}[Ru(NH_3)_4(NO_2)(L)]^{+} + H_2O$$

The equilibrium constant (K_{eq}) of this reaction was already determined for the complex under study, *trans*-$[Ru(NH_3)_4(NO)(py)]^{3+}$ as $K_{eq} = 2.2 \times 10^5$ L^2 mol^{-2} in references.[2-4]

Furthermore, we have titrated an aqueous solution of *trans*-$[Ru(NH_3)_4(NO)(py)]^{3+}$ and followed the spectral changes by UV-Vis spectroscopy. The main feature is the formation of an intense band at 380 nm, assigned to *trans*-$[Ru(NH_3)_4(NO_2)(py)]^{+}$, which is also observed for other *trans*-$[Ru(NH_3)_4(NO_2)(L)]^{+}$ complexes:

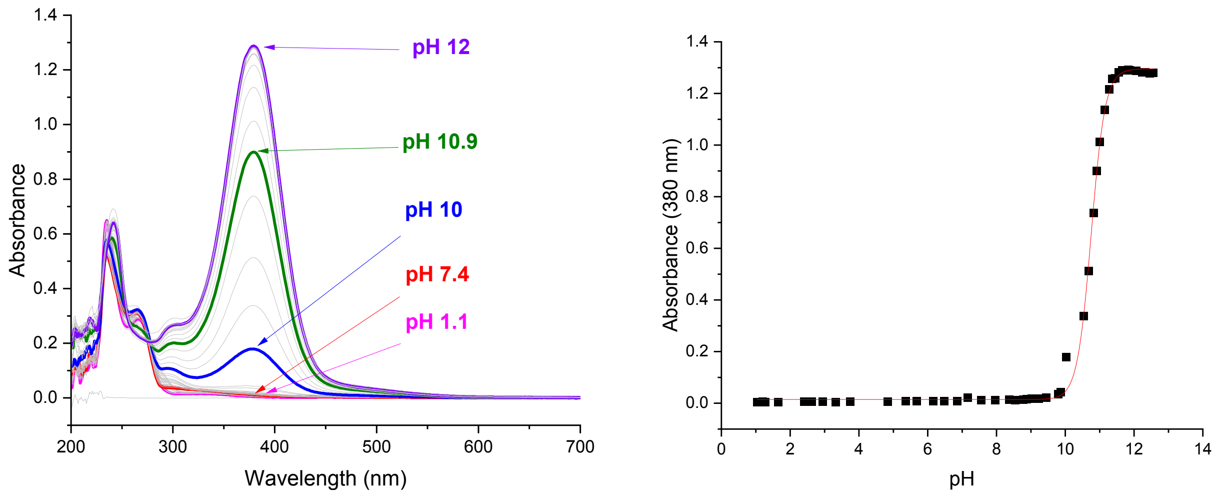

Figure S4. **(A)** titration of complex *trans*-$[Ru(NH_3)_4(NO)(py)]^{3+}$ with sodium hydroxide solution. **(B)** Absorbance at 380 nm vs. pH.

This result clearly shows that at pH 7.4 there is no significant formation of the respective nitro complex *trans*-$[Ru(NH_3)_4(NO_2)(py)]^+$. Significant amounts of the nitro complex only start to be formed at pH close to 10.

Furthermore, we have synthesized the *trans*-$[Ru(NH_3)_4(NO_2)(py)][PF_6]$, and the FT-IR spectra of this complex is shown in the Figure S5:

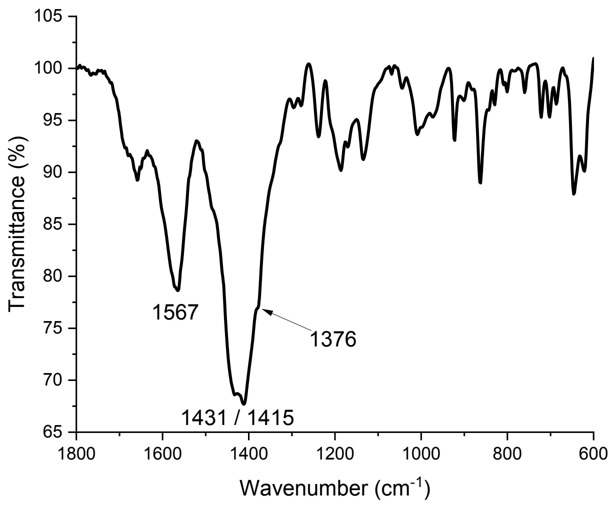

Figure S5. **(A)** FT-IR of the nitro complex *trans*-$[Ru(NH_3)_4(NO_2)(py)][PF_6]$.

Typical nitro complexes exhibit $\nu_a(NO_2)$ and $\nu_s(NO_2)$ in the 1470–1370 and 1340–1320 cm^{-1} regions, respectively.[5] This is consistent with the bands observed for nitro complex that we isolated, the *trans*-$[Ru(NH_3)_4(NO_2)(py)][PF_6]$. The most intense bands of this complex are observed at 1567,1431 and 1415 cm^{-1}. Below is shown the spectroelectrochemistry result in the 1500–1275 cm^{-1} range for the nitrosyl complexes *trans*-$[Ru(NH_3)_4(^{14}NO)(py)]^{3+}$ and the ^{15}N-labeled *trans*-$[Ru(NH_3)_4(^{15}NO)(py)]^{3+}$. We

do not observe any band centered at 1567,1431 and 1415 cm^{-1} region, confirming that the nitro complex Ru-NO_2 *trans*-$[Ru(NH_3)_4(NO_2)(py)]^+$ is not formed.

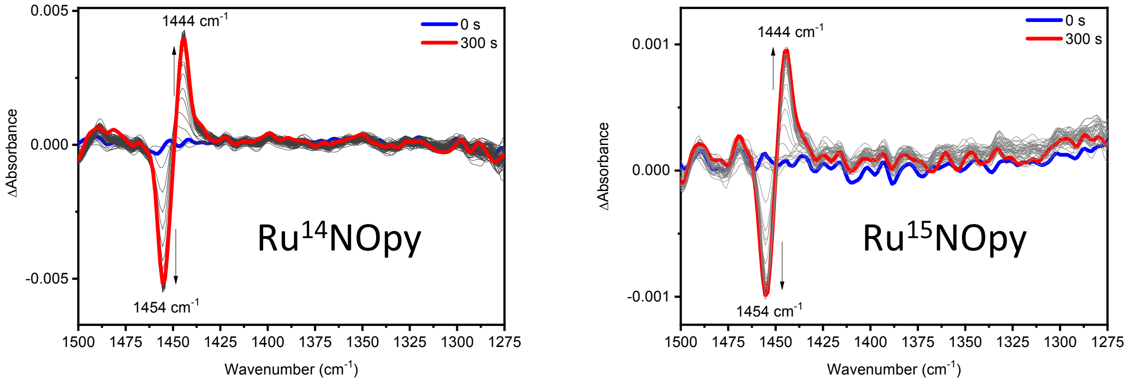

Figure S6. FT-IR spectroelectrochemistry of (A) RuNOpy and (B) 15N-labeled $Ru^{15}NOpy$. Spectral region of 1500-1275 cm^{-1}. Each FT-IR spectrum was recorded at 3.5 s time intervals.

Based on the results above, we assure that the nitro complex *trans*-$[Ru(NH_3)_4(NO_2)(py)]^+$ was not formed under our experimental conditions.

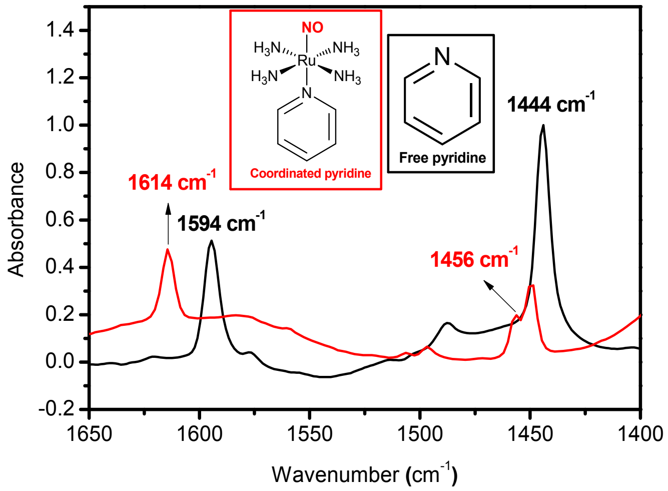

Figure S7. Solution ATR-IR spectra of RuNOpy (10 x 10^{-3} mol L^{-1}, red) and pyridine (10 x 10^{-3} mol L^{-1}, black) in phosphate buffer solution prepared using D_2O (pH = 7.4 ± 0.1; μ = 0.262 mol L^{-1}).

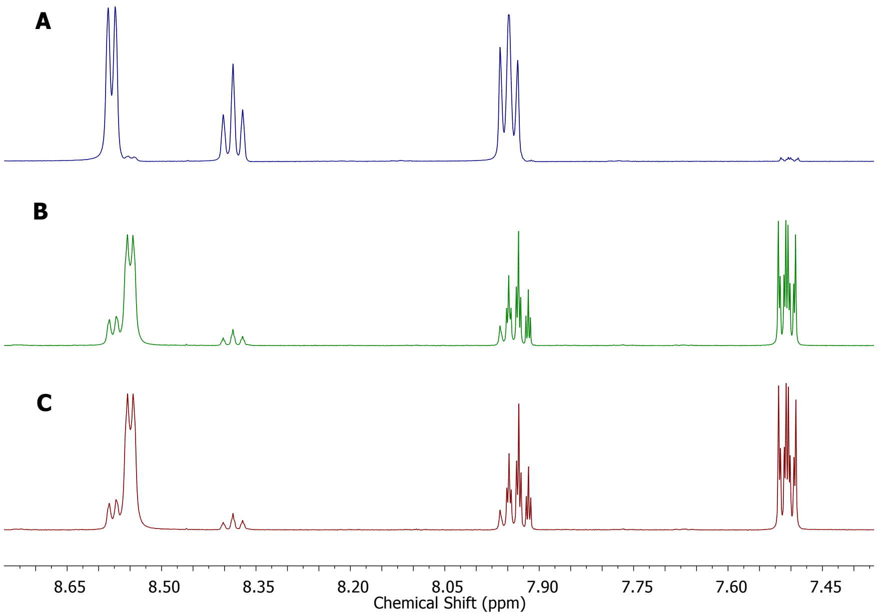

Figure S8. Labilization of pyridine after electrolysis of RuNOpy (5 x 10^{-3} mol L^{-1}), in phosphate buffer solution prepared with D_2O (pH = 7.4 ± 0.1, μ = 0.262 mol L^{-1}). **(A)** ^{1}H-NMR spectrum before the electrolysis. **(B)** ^{1}H-NMR spectrum after exhaustive electrolysis. Applied potential: -0.30 V (vs. $Ag/AgCl_{sat}$) for 30 minutes. **(C)** ^{1}H-NMR spectrum of the electrolyzed solution, after addition of pyridine solution.

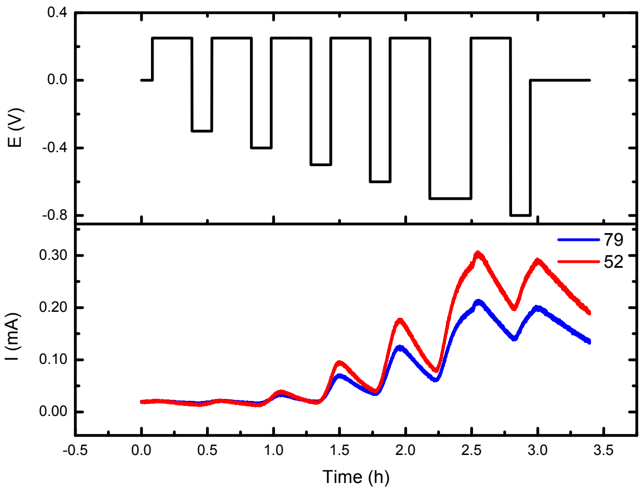

Figure S9. EC-MS results correlating applied potential and detected m/z related to fragments of pyridine, upon reducing RuNOpy in phosphate buffer solution (pH = 7.4 ± 0.1; μ = 0.262 mol L^{-1}).

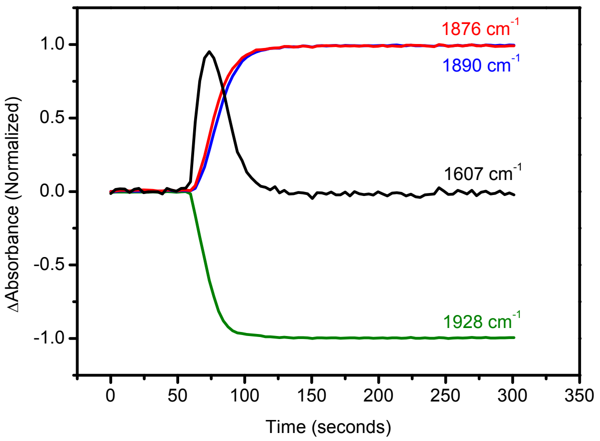

Figure S10. Time evolution of the signals observed during FT-IR spectroelectrochemical experiment using RuNOpy, by applying -0.20 V (*vs.* Ag/AgCl$_{sat}$), in phosphate buffer solution prepared using D_2O (pH = 7.4 ± 0.1; μ = 0.262 mol L^{-1}).

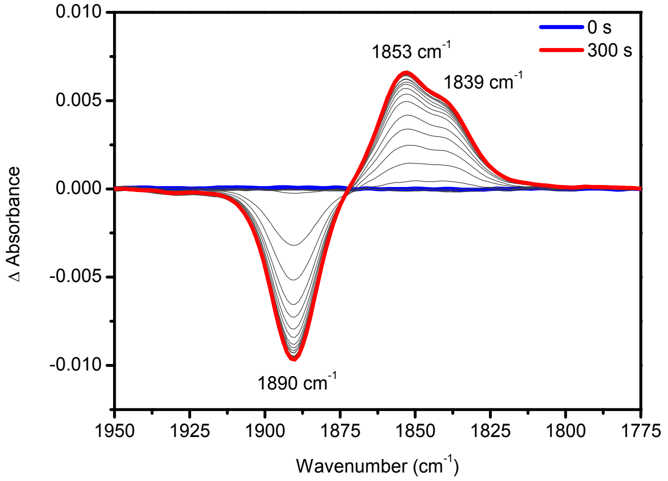

Figure S11. FT-IR spectroelectrochemistry of $Ru^{15}NOpy$ (5 x 10^{-3} mol L^{-1}) upon applying -0.20 V (*vs.* Ag/AgCl$_{sat}$), in phosphate buffer solution prepared using D_2O (pH = 7.4 ± 0.1; μ = 0.262 mol L^{-1}). Spectral region: 1950-1775 cm^{-1}. Each FT-IR spectrum was recorded at 3.5 s time intervals.

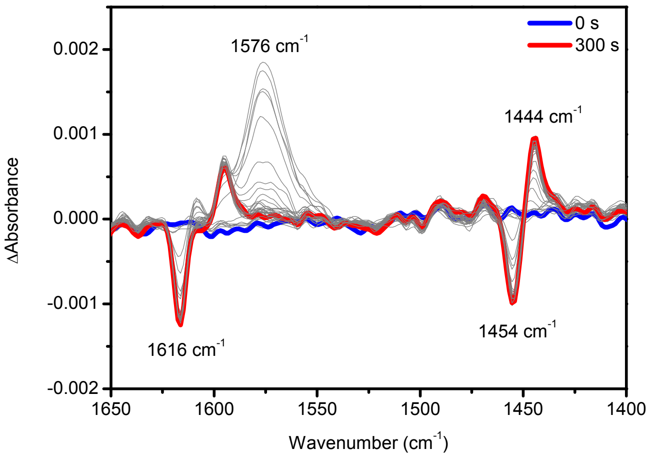

Figure S12. FT-IR spectroelectrochemistry of $Ru^{15}NOpy$ (5×10^{-3} mol L^{-1}) upon applying -0.20 V (*vs.* $Ag/AgCl_{sat}$), in phosphate buffer solution prepared using D_2O (pH = 7.4 ± 0.1; μ = 0.262 mol L^{-1}). Spectral region: 1650-1400 cm^{-1}. Each FT-IR spectrum was recorded at 3.5 s time intervals.

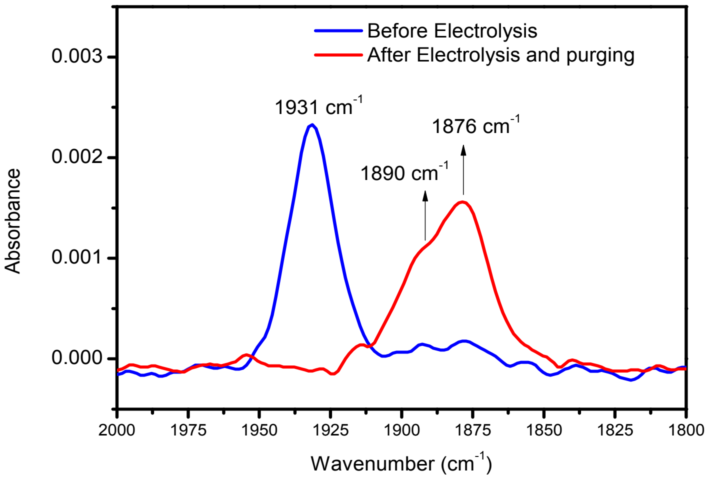

Figure S13. ATR-IR spectrum of a solution of RuNOpy before exhaustive electrolysis (blue) and solution IR spectrum after exhaustive electrolysis and purging (red). Applied potential: -0.30 V (*vs.* $Ag/AgCl_{sat}$) for 30 minutes.

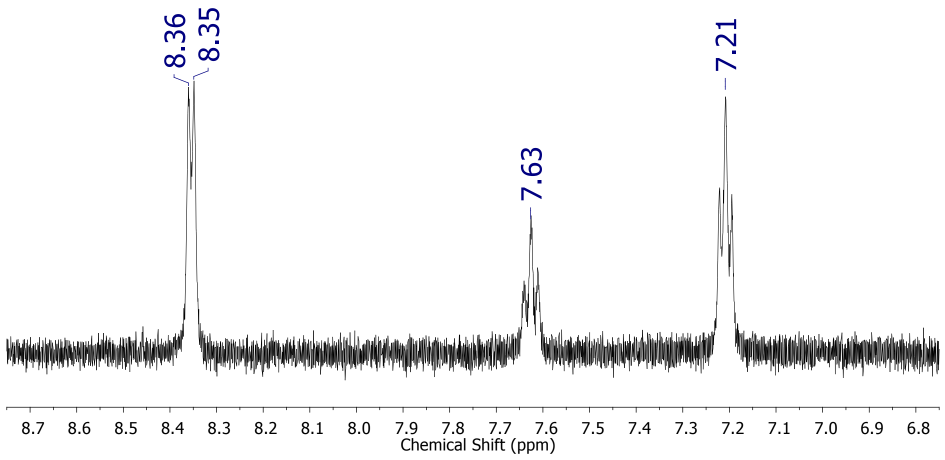

Figure S14. ^{1}H-NMR spectrum of complex *trans*-$[Ru(H_2O)(NH_3)_4(py)]^{2+}$ in phosphate buffer solution prepared using D_2O (pH 7.4 ± 0.1, μ = 0.262 mol L^{-1}).

Table S1. Experimental and calculated ν(NO) for the nitrosyl complexes

Complex	ν(NO) experimental	ν(NO) calculated
trans-$[Ru(NO^+)(NH_3)_4(py)]^{3+}$ (RuNOpy)	1928 cm^{-1}	1953 cm^{-1}
trans-$[Ru(NO^\bullet)(NH_3)_4(py)]^{3+}$ ($RuNO^\bullet py$)	1607 cm^{-1}	1675 cm^{-1}
cis-$[Ru(NO^+)(NH_3)_4(OH)]^{2+}$ *cis*-RuNO(OH)	1890 cm^{-1}	1923 cm^{-1}
trans-$[Ru(NO^+)(NH_3)_4(OH)]^{2+}$ *trans*-RuNO(OH)	1876 cm^{-1}	1898 cm^{-1}

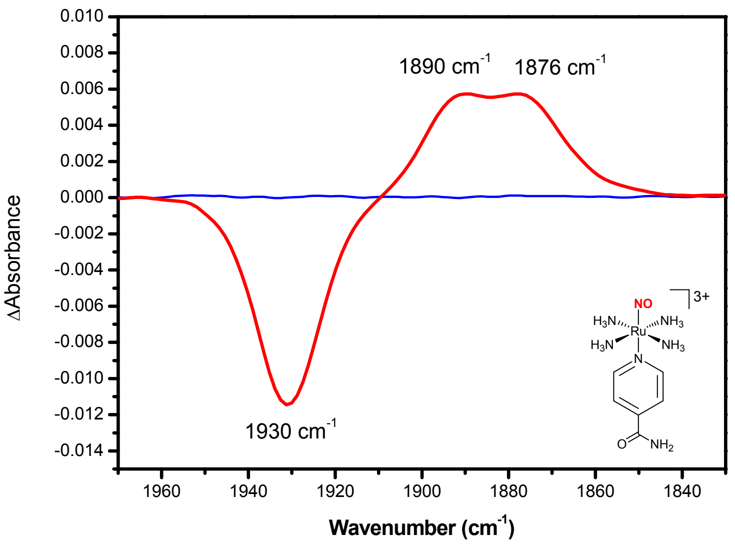

Figure S15. FT-IR spectroelectrochemistry of *trans*-[Ru(NO$^+$)(NH$_3$)$_4$(isn)]$^{3+}$ (5 x 10^{-3} mol L^{-1}) upon applying -0.25 V (vs. Ag/AgCl$_{sat}$), in phosphate buffer solution (pH = 7.4 ± 0.1; μ = 0.262 M). The red spectrum was recorded at 5 min of applied potential.

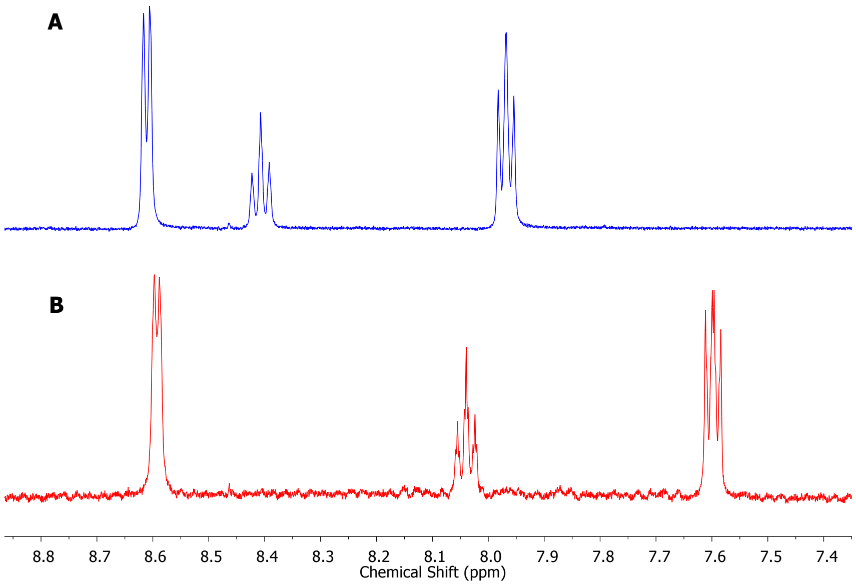

Figure S16. Labilization of pyridine after electron transfer chain reaction experiment for complex RuNOpy (5 x 10^{-3} mol L^{-1}) in phosphate buffer solution prepared with D$_2$O (pH = 7.4 ± 0.1, μ = 0.262 mol L^{-1}). **(A)** ^{1}H-NMR spectrum before reduced with ZnHg amalgams. **(B)** ^{1}H-NMR spectrum after exposing 50% of the solution to ZnHg amalgams, allowing the electron transfer chain reaction.

Table S2. XYZ coordinates for optimized structure of *trans*-$[Ru(NO^{+})(NH_3)_4(py)]^{3+}$ (RuNOpy).

	x	y	z
Ru	0.83033	0.00003	0.00000
O	3.74772	-0.00011	0.00003
N	-1.30032	0.00003	-0.00002
N	0.75206	-1.49937	1.57208
H	1.41575	-1.32431	2.34109
H	0.96533	-2.45966	1.26519
H	-0.17649	-1.54757	2.01563
N	0.75213	1.49994	1.57138
H	1.41658	1.32567	2.33993
H	-0.17610	1.54767	2.01564
H	0.96449	2.46025	1.26391
N	2.60897	-0.00005	0.00001

C	-4.11671	-0.00005	0.00001
H	-5.20929	-0.00009	0.00002
C	-2.01058	-1.16904	0.00003
H	-1.44729	-2.10365	0.00002
C	-3.40036	1.20277	-0.00006
H	-3.91018	2.16838	-0.00010
C	-2.01066	1.16906	-0.00007
H	-1.44742	2.10374	-0.00012
C	-3.40029	-1.20284	0.00005
H	-3.91004	-2.16848	0.00009
N	0.75214	-1.49987	-1.57156
H	1.41663	-1.32556	-2.34007
H	0.96442	-2.46022	-1.26422
H	-0.17610	-1.54748	-2.01585
N	0.75216	1.49933	-1.57190
H	1.41542	1.32389	-2.34120
H	-0.17656	1.54806	-2.01502
H	0.96615	2.45948	-1.26506

Table S3. XYZ coordinates for optimized structure of *trans*-[Ru(NO•)(NH_3)$_4$(py)]$^{3+}$ (RuNO•py).

	x	y	z
Ru	0.83341	-0.04923	-0.08149
O	3.60423	0.29599	0.55356
N	-1.37580	0.01599	-0.01135
N	0.80330	-1.33434	1.65871

H	1.48134	-1.04000	2.37412
H	1.04381	-2.31519	1.46653
H	-0.11239	-1.35420	2.12556
N	0.97757	1.62560	1.26876
H	1.92620	1.67304	1.67049
H	0.32895	1.58639	2.06523
H	0.82563	2.55059	0.84648
N	2.68451	-0.08513	-0.08955
C	-4.18667	0.09416	0.16184
H	-5.27526	0.12450	0.22873
C	-2.11259	-1.12089	0.09717
H	-1.56365	-2.06442	0.10534
C	-3.44343	1.27167	0.04776
H	-3.92818	2.24832	0.02194
C	-2.05615	1.19184	-0.03132
H	-1.46181	2.10389	-0.11404
C	-3.50157	-1.12320	0.18763
H	-4.03322	-2.07177	0.27203
N	0.77577	-1.74259	-1.45276
H	1.65039	-1.77663	-1.99319
H	0.70530	-2.66961	-1.01431
H	0.01083	-1.70821	-2.13817
N	0.76620	1.25684	-1.81672
H	1.33085	0.89267	-2.59429
H	-0.18427	1.37934	-2.18830
H	1.13333	2.20344	-1.65503

Table S4. XYZ coordinates for optimized structure of *cis*-$[Ru(NO^+)(NH_3)_4(OH)]^{2+}$ (*cis*-RuNO(OH)).

	x	y	z
Ru	0.05916	-0.00883	-0.00164
O	-2.82190	0.06196	-0.29346
N	0.28498	-1.99225	-0.82286
H	-0.60574	-2.43384	-1.08507
H	0.77869	-2.68794	-0.24931
H	0.81752	-1.89039	-1.69951
O	0.65536	0.72216	-1.74217
H	0.12997	0.49298	-2.53209
N	-1.67865	0.01985	-0.19279
N	-0.09739	-0.76173	2.08871
H	-0.47889	-0.05984	2.73567
H	-0.71688	-1.57735	2.17937
H	0.79853	-1.05260	2.49900
N	0.09248	2.08322	0.48543
H	-0.80580	2.49098	0.77306
H	0.78139	2.40787	1.17467
H	0.33732	2.49674	-0.43138
H	2.71740	-0.86352	0.29995
N	2.23199	0.03893	0.21736
H	2.51152	0.44233	-0.69313
H	2.63044	0.63392	0.95481

Table S5. XYZ coordinates for optimized structure of *trans*-[Ru(NO$^+$)(NH$_3$)$_4$(OH)]$^{2+}$ (*trans*-RuNO(OH)).

	x	y	z
Ru	0.09319	-0.00001	-0.01665
O	-2.80666	-0.00016	0.37228
N	-0.00707	1.54960	-1.51981
H	-0.66741	2.30523	-1.29440
H	-0.26852	1.23994	-2.46489
H	0.91996	1.98801	-1.60865
N	0.53696	1.50468	1.45516
H	0.17230	2.44635	1.26383
H	1.56677	1.56831	1.44528
H	0.24840	1.29176	2.41843
N	-1.66012	-0.00014	0.25927
N	-0.00667	-1.55024	-1.51918
H	-0.66676	-2.30597	-1.29339
H	-0.26834	-1.24105	-2.46437
H	0.92047	-1.98841	-1.60797
N	0.53736	-1.50399	1.45576
H	0.17276	-2.44579	1.26497
H	1.56717	-1.56750	1.44578
H	0.24893	-1.29061	2.41897
H	2.48107	0.00001	-1.06013
O	2.01537	0.00024	-0.20344

References

(1) Laverman, L. E.; Ford, P. C. Mechanistic Studies of Nitric Oxide Reactions with Water Soluble Iron(II), Cobalt(II), and Iron(III) Porphyrin Complexes in Aqueous Solutions: Implications for Biological Activity. *J. Am. Chem. Soc.* **2001**, *123* (47), 11614–11622. DOI: 10.1021/ja0113910.

(2) Borges, S. da S. S.; Davanzo, C. U.; Castellano, E. E.; Z-Schpector, J.; Silva, S. C.; Franco, D. W. Ruthenium Nitrosyl Complexes with N-Heterocyclic Ligands. *Inorg. Chem.* **1998**, *37* (11), 2670–2677. DOI: 10.1021/ic951563s.

(3) Gomes, M. G.; Davanzo, C. U.; Silva, S. C.; Lopes, L. G. F.; Santos, P. S.; Franco, D. W. Cis- and Trans-Nitrosyltetraammineruthenium(II). Spectral and Electrochemical Properties and Reactivity. *J. Chem. Soc., Dalton Trans.* **1998**, No. 4, 601–608. DOI: 10.1039/a708164b.

(4) Roncaroli, F.; Ruggiero, M. E.; Franco, D. W.; Estiú, G. L.; Olabe, J. A. Kinetic, Mechanistic, and DFT Study of the Electrophilic Reactions of Nitrosyl Complexes with Hydroxide. *Inorg. Chem.* **2002**, *41* (22), 5760–5769. DOI: 10.1021/ic025653q.

(5) Nakamoto, K. *Infrared and Raman Spectra of Inorganic and Coordination Compounds*, 3rd ed.; Wiley: New York, 1978.
